# Echoes of the Month

**Published:** 1924-04

**Authors:** 


### ECHOES OF THE MONTH.

### WHITE BREAD BAD FOR TEETH.

Sir Harry Baldwin, Surgeon-Dentist to the King, lecturing before the People's League of Health, drew attention to the chief methods of avoiding the decay of the teeth. These, he insisted, were to ensure an active circulation of blood in the gums by means of a vigorously but discreetly used tooth-brush, that need not be of too stiff bristles. Hygiene of the mouth was all important, but he did not think dentrifices assisted in this. He advocated the daily use of a simple salt solution in preference, and adequate dental inspection at intervals. The decay of teeth was the result of the lack of certain vitamines in food. Especially was this the case with the poorer classes, with whom bread was the staple article of diet. The trouble was due to the fact that the bread they used was made of ordinary white flour from which all the vitamines had been removed in the process of whitening, and not only the vitamines, but most of its proteins and valuable mineral salts. Accordingly he would like to see wholemeal bread in general use, as it contained to the full vitamines, proteins and salts.

### POISON AS A FINE ART.

Dr. H. Leighton Jones, Government medical and health officer of the Northern Territory, who has arrived in Brisbane after a tour of the islands between Darwin and Singapore in a 15-ton auxiliary schooner, gives a remarkable description of the Dutch island of Adinara, a few miles distant across the straits. Murders, he said, were a daily occurrence, and, according to a belief of the natives, no resident of the island died naturally, death usually being attributed to witchcraft by members of some adjacent tribe. The consequence was that it was a common thing for whole families to be annihilated simply because someone had died, the death being attributed to their machinations. Poisoning is a fine art among the Malays. They can limit their murders to days, months, or even years. Many Rajahs and even Dutch officials never partake of food until one hour has elapsed after the cook has eaten of the dishes.

### HEART BEATING AFTER DEATH.

Addressing the Oxford Junior Scientific Club, Sir Bernard Spilsbury said that it was possible to be led astray by the very signs on which the expert relied. If the beating of the heart was the sign of life, the experts were hard put to it sometimes to say when a man was dead. He remembered a French case which was very much to the point, in that a man was properly and effectively guillotined, and his heart continued to beat regularly for an hour afterwards. This seemed somewhat incredible, but it was made possible by the wonderful elasticity of the veins. The head was cut off cleanly, very low in the neck, and the veins simply retired into the chest, joined, and continued to work the heart until the supply of oxygen in the body had been exhausted. It was particularly difficult to say whether or not a man was dead after electrocution, and in many accidents which occurred in connection with electricity apparently dead people were allowed to become really dead because it was so hard to tell. He was glad that instructions had now been issued that artificial respiration must continue for a long period in cases of electrocution, whether there were any apparent results or not.

### DEAN OF ST. PAUL'S ON RACE DEGENERATION

"The upper middle class in this country and the United States are dying out," said the Dean of St. Paul's to the Charity Organisation Society. The lowest birth-rate is among the doctors, the teaching profession, and ministers of religion. You could not find three professions containing a greater amount of intelligence, good physique, and good character, and it is they who are dying out. The process has been accelerated since the war because of the crushing burden of taxation laid on the professional classes and the terrible loss during the war among the officer class. The feeble-minded were about 50 per cent. more prolific than the normal, and since 1859 the insane had increased in numbers by 230 per cent. He suggested the following remedies: Money spent on education should be deducted from the taxable income of the parent. Death duties should be computed according to the distribution of the bequests. A fortune left entirely to one son should be more heavily taxed than the same amount distributed among several children. The State should undertake to educate a certain number only in each family. This would tend to restrict the birth-rate by making the father educate children in excess of this number at his own cost. Absolutely reckless propagation of the unfit should be checked.

### THE INSANITARY HOUSE OF COMMONS.

Dr. Haden Guest, M.P., in an address at the Institute of Hygiene, Devonshire Street, W., recently, on "The Health of the Shop Assistant," remarked that conditions of living and working in the House of Commons were very insanitary. "It is," he said, "one of the worst workships that I have ever come across. That is partly because there have not been enough people in Parliament with medical knowledge."

### POOR JOHNNY PENCIL.

To emphasize the dangers (from the standpoint of health) inherent in pencil chewing, or in putting any object which may not be sterile in the mouth, Chicago children are shown little Johnny Pencil, a lead pencil such as may be purchased in many novelty stores which has the face of a little boy at one end. When the children see his battered little face twisted out of shape and mutilated, they learn the lesson.

### WHITE MEN TURN BLACK.

Another case has come to light at Hull of a white man turning black through a mysterious disease which doctors and specialists have not yet been able to identify. The disease is extremely rare. Investigations by Hull doctors go to show that besides the two cases now at Hull, only one other case has been discovered, which was treated at Guy's Hospital, London, the patient being known as "Blue May." There is an old Danish seaman lying at the Sculcoates (Hull) Workhouse Infirmary who, during the last six months, has changed a blue-black colour from head to foot. A representative of the *Yorkshire Post* found another man who has been in a similar condition for over five years, and, although he has been under several London specialists and local doctors, he still remains a curious slate-grey which, at night, deepens to a deep ebony colour, so that he almost resembles a negro. This man was for thirty-two years employed as a marine fireman, and it has been suggested that the intense heat was the cause of his condition.

### INSURANCE AGAINST DOCTORS' RISKS.

London underwriters are now being asked to cover the risk of loss that may be suffered by medical practitioners in wrongfully certifying individuals as insane. It is recognised that any policies issued for this purpose would need to be worded carefully, and a form of suitable wording is being considered. Probably such insurances would only take effect if the loss amounted to more than a certain figure, and the total sum covered by each policy might be large. It is understood that many insurances have in the past been issued to cover medical men and dental surgeons against claims by patients for mistakes made in the course of treatment. Protection is also provided by medical societies.

### THE COLOUR OF BLOOD.

Lecturing before the Royal Institution Professor Barcroft pointed out that blood is not necessarily red. We could not at present tell why it should not have been green. Indeed, among higher animals he had seen occasional specimens which were shaping in that direction. Once, for instance, he saw a rat with brown blood. The cuttlefish had blue blood, or blood which was sometimes blue. Just as human blood changed its colour each time it traversed the lung, so did the cuttlefish each time it was driven through that creature's gills. Our blood was purple when it reached our lungs; red when it left them. Cuttlefish blood was colourless when it passed to the gills, blue when it left them. Again there was a starfish in whose blood might be seen colours of the most diverse type—brown, purple, green, lemon yellow and indigo blue. The brown colour became green when it lost its oxygen. These colours enabled an animal to grow large owing to their power of carrying oxygen. Thus insects which had dropped the colour from their blood remained small. The molluscs singled out a blue pigment for their use—a colour dependent on the copper which it contained. These creatures reached their highest development in the massive, but almost mindless, cuttlefish. The higher animals (vertebrates) chose iron-containing colours, and had become "the lords of creation."

### VILLAGE COMMUNITY LIFE FOR THE INSANE

At Bangour, between Edinburgh and Linlithgow, unde- the Edinburgh Board of Control, there is a village community where mental cases are cared for without laying stress on the abnormality of the patients or suggesting to their minds those prohibitions which, in other institutions, are not conducive to a patient's recovery. The buildings, which are dotted about in a spacious manner, cover about 200 acres, the rest of the ground, extending to about 1,000 acres, being farmed. The community contains some seven miles of roads. The approach to its headquarters, through well-kept lawns and neat drives, is striking and entirely devoid of the forbidding aspect which high prison-like walls give many another institution. There is, in fact, not even a gate to prevent the inmates from escaping, and this fact, typical of the general policy pursued, is said to have resulted in far fewer attempts to escape than is usual in asylums. The community is to a large extent self-supporting, as far as the necessities of life go. The patients assist on the farm—in milking and dairying operations and in its general work.

### DANGERS OF GLASS JAM JARS.

Writing in *Municipal Engineering and Sanitary Record*, a member of a big firm of jam makers confirms all that has been said about the dangers attending the use of glass containers for jam, jelly and marmalade. "I leave the doctors to settle among themselves what happens after the glass is swallowed; but common sense tells me that a diet interspersed with glass spicules must sometimes be harmful. The bursting of glass bubbles inside the jar is a source of trouble, but thin glass projections coming out from the 'seam' inside are, perhaps, worse. Glass jars are now manufactured by machinery in very large numbers, and when the two halves of the moulds do not fit tightly together glass is squeezed out in the form of thin plates. The hot jam causes them to crack, or, if they survive that ordeal, a metal spoon used in scooping out the jam will sometimes break them off. Some consignments have this defect to such an extent that we employ men to examine each jar, break off any excrescences they may find, remove the pieces, and blow out the glass dust. Since the use of glass became so popular for preserves, the manufacturers' responsibility and anxiety have greatly increased."

### THE CANCER PARASITE.

A special parasite which Dr. James Young, of Edinburgh University, believes he has obtained from cancerous tumours in man and in animals, and the remarkable results obtained when the micro-organism is injected artificially into a healthy animal, are the subject of an article in the *Empire Review*. The special parasite, Dr. Young claims, "can actually be seen growing out of the inner recesses of the cell into the artificial nutritive growths in which he has incubated the growths." He further claims that in its laboratory phase the "cancer parasite belongs to a familiar type of germ." He believes that it is probably, like the germs of some other diseases, almost ubiquitous. When the cancer micro-organism is injected artificially into a healthy animal it "goes straight for widespread parcels of primitive cells and provokes in them an active multiplication similar in its intimate features to an ordinary cancer." The disease which Dr. Young has thus produced in the laboratory resembles a disease in man (leukæmia) which has for long been considered by many doctors to be a kind of cancer. Dr. thoung's experiment on an animal raises no less an issue than Ye possibility of the "wholesale protection of man himself against the ravages of this dire disease."

